# Optic Nerve Head Quantification in Idiopathic Intracranial Hypertension by Spectral Domain OCT

**DOI:** 10.1371/journal.pone.0036965

**Published:** 2012-05-15

**Authors:** Falko Kaufhold, Ella Maria Kadas, Christoph Schmidt, Hagen Kunte, Jan Hoffmann, Hanna Zimmermann, Timm Oberwahrenbrock, Lutz Harms, Konrad Polthier, Alexander U. Brandt, Friedemann Paul

**Affiliations:** 1 NeuroCure Clinical Research Center and Experimental and Clinical Research Center, Charité – Universitätsmedizin Berlin and Max Delbrück Center for Molecular Medicine, Berlin, Germany; 2 Institute of Neuroradiology, Charité – Universitätsmedizin Berlin, Berlin, Germany; 3 Department of Neurology, Charité – Universitätsmedizin Berlin, Berlin, Germany; 4 Clinical and Experimental Multiple Sclerosis Research Center, Charité – Universitätsmedizin Berlin, Berlin, Germany; 5 Mathematical Geometry Processing Group, Freie Universität Berlin, Berlin, Germany; Hannover Medical School, Germany.

## Abstract

**Objective:**

To evaluate 3D spectral domain optical coherence tomography (SDOCT) volume scans as a tool for quantification of optic nerve head (ONH) volume as a potential marker for treatment effectiveness and disease progression in idiopathic intracranial hypertension (IIH).

**Design and Patients:**

Cross-sectional pilot trial comparing 19 IIH patients and controls matched for gender, age and body mass index. Each participant underwent SDOCT. A custom segmentation algorithm was developed to quantify ONH volume (ONHV) and height (ONHH) in 3D volume scans.

**Results:**

Whereas peripapillary retinal nerve fiber layer thickness did not show differences between controls and IIH patients, the newly developed 3D parameters ONHV and ONHH were able to discriminate between controls, treated and untreated patients. Both ONHV and ONHH measures were related to levels of intracranial pressure (ICP).

**Conclusion:**

Our findings suggest 3D ONH measures as assessed by SDOCT as potential diagnostic and progression markers in IIH and other disorders with increased ICP. SDOCT may promise a fast and easy diagnostic alternative to repeated lumbar punctures and could therefore ease monitoring of treatment or disease progression.

## Introduction

Idiopathic intracranial hypertension (IIH), also known as pseudotumor cerebri (PTC), is a clinical syndrome of unknown etiology characterized by increased intracranial pressure (ICP) which typically affects young, obese women of childbearing age [1]–[5]. Clinical symptoms include headache, visual disturbances, pulsating tinnitus, photopia, eye pain, diplopia and nausea. Papilledema with subsequent visual field loss is the most feared clinical consequence, which mainly determines the therapy and outcome of the syndrome [6]–[9]. However, although detection of papilledema by an experienced ophthalmologist is a powerful tool in primary diagnosis, it remains a limited method in producing quantitative data to evaluate longitudinal optic disc changes in patients with IIH [10], [11].

Optical coherence tomography (OCT) is a non-invasive imaging method, which creates in vivo cross-sectional patterns of the retina [12]. In recent years, OCT has become a valuable tool for assessing retinal axonal damage in several neurological diseases such as optic neuritis, multiple sclerosis, neuromyelitis optica, spinocerebellar ataxia, and Parkinson's disease [13]–[20]. Moreover, OCT is associated both with morphologic and metabolic changes in brain, thus providing an easy accessible window into the brain in neurologic diseases [21]–[23].

Few studies investigated OCT in IIH using the retinal nerve fiber layer thickness (RNFLT) in a peripapillary ring scan as outcome parameter. Patients with newly diagnosed IIH presented RNFL thickening compared to healthy controls, which decreased after three months under IIH treatment, thus proposing RNFLT as a potential longitudinal measure [24]–[26]. Subsequently it was shown, that peripapillary RNFLT swelling due to increased ICP correlates well with optic disc elevation or papilledema in funduscopy [27]. However, all these studies used 2D time domain OCT that does not allow direct 3D measurement of the optic nerve head (ONH). Consequently, the peripapillary RNFLT measurement is only a rough estimate of ONH changes as it is located at the marginal zone of the pathologic process.

Next generation 3D spectral-domain OCT (SDOCT) potentially allows direct ONH quantification and has therefore been proposed for evaluation of papilledema in IIH [28]. However, ONH changes in patients with IIH (i.e. swelling) impede the use of existing algorithms focusing on other diseases [29]. For this reason we developed a novel custom segmentation algorithm using an extension of the retinal pigment epithelium through the ONH as reference line, which enabled us to automatically assess ONH volume and shape in IIH patients that should also be applicable in other diseases with elevated ICP and optic disc swelling.

Next, in a prospective cross-sectional pilot study we aimed to quantify ONH differences by SDOCT between IIH patients and matched controls. Lastly, we investigated a possible association between SDOCT measures and clinical data which would be relevant for the question whether ONH quantification may serve as a tool for monitoring disease progression and therapeutic effects in IIH.

## Methods

### Patients and Participants

The study included patients with a minimum age of 18 years and a definite diagnosis of IIH according to the modified Dandy criteria [30]. Patients were consecutively recruited from the neurology outpatient clinic at the Charité- Universitätsmedizin Berlin between June 2010 and July 2011. Exclusion criteria were systemic conditions or medication affecting ICP other than IIH related medication, pregnancy or postpartum period, surgical interventions affecting cerebro-spinal fluid circulation (e.g. shunting procedures or fenestration of the optic nerve sheath) and an ophthalmological disease which could influence OCT imaging (i.e. glaucoma).

All patients received a complete neurological examination supervised by a board certified neurologist. The following data were compiled: disease duration, current body mass index (BMI), most recent lumbar puncture opening pressure (latest ICP), current medical treatment and current IIH symptoms (i.e. headache, visual obscurations, tinnitus, dizziness, nausea). For measurement of ICP patients were lying on their right side in a fetal position. After the subarachnoid space was punctured, cerebrospinal fluid opening pressure was determined by the means of a column manometer. A cohort of age, gender and BMI matched controls was recruited among employees and obese patients from the psychosomatic medicine department of the Charité- Universitätsmedizin Berlin, which were treated only for adiposity. Symptoms of increased ICP as well as a diagnosis of IIH were ruled out clinically in the control group by board certified neurologists. The same exclusion criteria and examinations of the patient group applied to the control group (CG). The study was approved by the local ethics committee of the Charité- Universitätsmedizin Berlin and was conducted in accordance to the Declaration of Helsinki in its currently applicable version, the guidelines of the International Conference on Harmonisation of Good Clinical Practice (ICH-GCP) and the applicable German laws. All participants gave informed written consent.

### Optical Coherence Tomography

RNFLT, total macular volume (TMV) and 3D ONH scans were obtained using a spectral-domain OCT (Heidelberg Spectralis SDOCT, Heidelberg Engineering, Germany, Spectralis software version 5.3.3.0, Eye Explorer software 1.6.4.0) for each eye of the patients and matched controls. RNFLT was measured using a 3.4 mm circular scan around the ONH with the device's standard protocol and segmentation algorithm with activated eye tracker (TrueTrack®) and the maximum number of averaging frames in ART–MEAN mode was tried to achieve. TMV was measured by a custom protocol which generated 61 slices (B-scans) focusing the fovea centralis with a scanning angle of 30°×25° and a resolution of 768 A-scans per B-scan. TMV was calculated by estimating the distance between the inner limiting membrane and the Bruch-membrane in a cylinder with 6 mm in diameter using the device software's segmentation algorithm. The 3D ONH scan was performed using a custom protocol with 145 slices (B-scans), focusing the optic nerve head with a scanning angle of 15°×15° and a resolution of 384 A-scans per B-scan.

All scans were acquired by one of three experienced operators and were reviewed for sufficient signal strength, correct centering and segmentation by an independent operator not involved in the study. All operators were blinded towards patient and control groups; however, due to the nature of papilledema patients could be easily identified in most cases by ONH images alone.

### 3D Optic Nerve Head Analysis

**Figure 1:**
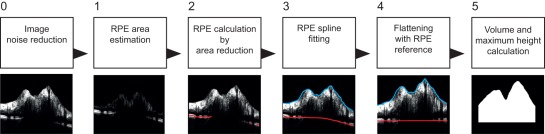
Overview of the custom segmentation process for assessing ONH volume and height in IIH patients. 0) First OCT B scans are cleaned from noise and smoothed, 1) then the area in which the RPE is expected is narrowed by removing bright upper layers of the scan, resulting in the brightest spots belonging most likely to the RPE. 2) From the A scan at the first quarter and the A scan at the last quarter of the B scan, the possible RPE area is further reduced by detecting the brightest spots of the image (red lines). 3) On the proposed RPE candidates a least square spline approximation is applied (red line), resulting in a hypothetical RPE through the ONH. The ILM is provided from the device's segmentation software (blue line). 4) The scans are flattened using the RPE as reference. 5) Finally volume and height are calculated in the newly defined area.

For the automatic assessment of ONH changes we developed a custom and fully automatic algorithm that measures the volume (ONHV) and maximum height (ONHH) of the disc edema. A detailed description of the algorithm including reliability and validation analyses is published elsewhere [31]. In brief, ONH OCT scans tend to have regions of strong varying intensity values caused by the edema. Additionally, scans are characterized by an increased intrinsic speckle noise making a reliable differentiation of intra retinal layers challenging to impossible. This algorithm identifies and uses two reference boundaries on each B-scan, the inner limiting membrane (ILM) and a hypothetical extension of the peripapillary retinal pigment epithelium (RPE) through the ONH. The edema is then defined as the area enclosed by these two layers. The ILM is provided in sufficient quality by the device's segmentation software. In order to compute the volume and maximal height of the edema the algorithm focuses on segmenting the RPE to create a base area for further calculation of both parameters. First an initial region that contains this layer is estimated. Starting from this region, unnecessary pixels are discarded. The RPE curve, describing the layer, is obtained by a fully automatic least square spline fitting of 2nd or 3rd order depending on the number of detected RPE segments. Furthermore, to account for the natural retinal curvature seen in SDOCT images, we performed an image flattening on each slice using again the RPE, followed by the final volume and height computation. For the volume measurement a threshold of 20 pixels was applied from the reference height computed at the right side and left side of each flattened B-scan. The area of the edema found on each B-scan multiplied by the special spacing was summed up to obtain the final volume. This threshold was selected to include most of the ONH and to provide a satisfying volume of the swelling in IIH as well as healthy controls. An overview of the segmentation algorithm is shown in figure 1.

For algorithm development and application, 3D images were exported from the SDOCT device and imported into Matlab using a custom import filter based on documentation provided by the device manufacturer. Development was performed using Matlab R2011A with additional library Spline (Mathworks, Ismaning, Germany).

### Statistical Analysis

Differences between the two study groups were analyzed by independent samples Mann-Whitney U test for age and BMI. For evaluation of the relationship between RNFLT, TMV, ONH parameters and clinical data, we performed generalized estimation equation (GEE) analyses with working correlation matrix structure “exchangeable” accounting for inter-eye/intra-patient dependencies. Clinical parameters (diagnosis and treatment) were used as independent and RNFLT, TMV or ONH parameters as dependent variables. Correlation of RNFLT with ONHH or ONHV was analyzed using Spearman's Rho.

For identifying a possible relationship of ICP with ONHH or ONHV we included only patients with an interval between ICP and OCT measurements of less than 24 months (n  = 11) and additionally weighted data in GEE models with ICP as the independent variable and either ONHH or ONHV as the dependent variable by factor *ω  = 100/(measurement distance in months+1)*. Hereby, OCT scans closer to the ICP measurements are more strongly weighted in the subsequent analysis, whereas scans with a longer interval are weighted less.

Reliability of ONHH and ONHV was determined using intra-class correlation coefficient on repeated measurements using the two-way mixed effects model where subject effects are random and measures effects are fixed.

RNFLT percentiles for comparison with normal values are given from the OCT device's internal normative database. All statistical analyses were performed with SPSS 19 (IBM SPSS Statistics Version 19, Release 19.0.0.1, IBM, Armonk, NY, USA). Statistical significance was established at p<0.05 in all analyses. All tests should be understood as exploratory data analysis, in that no previous power calculation and adjustments for multiple testing were performed.

## Results

### Cohort description

This study enrolled 37 eyes from 19 IIH patients and 38 eyes from 19 matched controls. One IIH eye had to be excluded due to newly diagnosed epiretinal membranes. Demographics of patients and controls are displayed in table 1. Patients and controls did not differ regarding gender, age and BMI. Headache (84%) was the most common symptom in IIH patients. Furthermore, all patients suffered from at least one of the following visual symptoms during the course of their disease: photopsia (79%), photophobia (58%), blurred vision (42%) and visual acuity loss (26%). They also described dizziness (58%), tinnitus (37%) and nausea (37%). Thirteen patients were treated pharmacologically with either acetazolamide (n  = 11), topiramate (n = 1) or furosemide (n = 1) at the time of OCT measurement.

**Table 1:** Demographic overview of IIH patients and controls.

	Parameter	Patients	Controls
N		19	19
Age (years)	Mean ± SD	38.0±13.8	37.6±12.9
	Min–Max	20–63	20–61
Gender (female/male)	N/N	17 / 2	17 / 2
BMI (kg/m^2^)	Mean ± SD	33.6±7.4	33.8±7.3
	Min–Max	24.1–54.6	24.8–48.1
Disease duration (months)	Mean ± SD	40±37	-
	Min–Max	0–115	-
Latest ICP (cmH_2_O)	Mean ± SD	28.6±6.3	-
	Min–Max	18.0–40.0	-
Time since last ICP (months)	Mean ± SD	20±20	-
	Min–Max	0–57	-

**Abbreviations**: IIH  =  idiopathic intracranial hypertension; ICP  =  intracranial pressure; SD  =  standard deviation; Min  =  minimum value; Max  =  maximum value.

### RNFLT and TMV differences

We found no significant differences in average RNFLT and TMV between IIH patients and CG (table 2). Although overall group differences were not significant, seven eyes from four IIH patients showed abnormally high RNFLT values above the 95^th^ percentile.

**Table 2:** Comparison of optical coherence tomography measurements between IIH patients and controls.

	Patients		Controls		GEE
	Mean ± SD	Min– Max	Mean ± SD	Min– Max	
RNFLT (µm)	99.1±18.1	58.4–155.5	99.2±8.0	79.9–111.9	P = 0.942
TMV (mm^3^)	8.48±0.40	7.72–9.01	8.67±0.38	7.87–9.28	P = 0.141
ONHV (mm^3^)	2.30±1.25	0.41–5.97	1.08±0.49	0.28–2.17	**p<0.001**
					B = 1.2; SE = 0.3
ONHH (mm)	0.42±0.07	0.34−0.64	0.40±0.03	0.34−0.46	P = 0.099

**Abbreviations**: RNFLT  =  retinal nerve fiber layer thickness; TMV  =  total macular volume; ONHV  =  optic nerve head volume; ONHH  =  optic nerve head height; SD  =  standard deviation; Min  =  minimum value; Max  =  maximum value; GEE  =  generalized estimating equation models analyses accounting for inter–eye/intra-patient dependencies; B  =  regression coefficient; SE  =  coefficient standard error; p  =  p value.

### Reliability of ONH measurements and quantification

To determine the reliability of ONHV and ONHH, each eye was measured three times in a row in 3 patients (6 eyes). Each measurement was then post-processed in a fully automated fashion using the presented algorithm. ICC for ONHV was 0.999 (p <0.001) and ICC for ONHH was 0.983 (p <0.001).

### ONH volume and height differences

**Figure 2:**
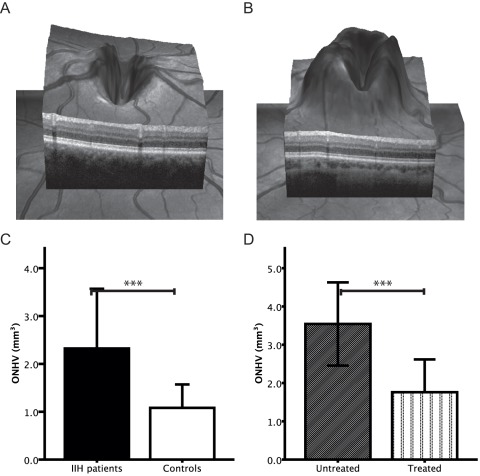
ONH volume and height differences between IIH patients and controls. A ) 3D spectral domain OCT ONH measurement from a matched control ONH **B**) 3D spectral domain OCT ONH measurement from an IIH patient with a diagnosed papilledema **C**) Groups differences in optic nerve head volume (ONHV) between IIH patients (black bar) and controls (white bar). **D**) Group difference in ONHV between medically untreated (gray bar) and treated (vertical lines bar) IIH patients. Error bars represent 1x standard deviation in figures c and d.  =  p <0.001.

ONHV in patients was increased compared to controls (p <0.001, table 2 and figure 2C). Furthermore, 18 eyes from 11 IIH patients showed an ONHV above the 95^th^ percentile of the CG (ONHV  = 2.09 mm^3^). ONHH did not differ between groups (table 2). Two sample 3D ONH scans from a control subject and an IIH patient are given in figure 2A and B. A receiver operating characteristic analysis (ROC) between IIH patients and controls presented a much higher area under the curve for ONHV (AUC  = 0.835) than for RNFLT (AUC  = 0.464).

We further divided the patient group into treated and untreated patients. Untreated patients (n  = 6) showed a higher ONHV than treated (n  = 13) patients (GEE, p<0.001, table 3 and figure 2D). Both medically treated and untreated patients had an increased ONHV compared to controls which did not hold true for ONHH. Additionally all but one eye from untreated patients showed an ONHV above the 95^th^ percentile of the CG. RNFLT analysis of the subgroups displayed also an increase for untreated patients (GEE, p = 0.03, table 3).

**Table 3:** Subgroup analysis of OCT measurements in medically treated and untreated IIH patients.

	untreated (n = 11 eyes)		treated (n = 26 eyes)		GEE
	Mean±SD	Min–Max	Mean ± SD	Min–Max	
RNFLT (µm)	112.5±19.2	90.3–155.5	93.4±14.5	58.4–120.5	**p = 0.030**
					B = 17.6; SE = 8.1
TMV (mm^3^)	8.41±0.34	7.8–8.9	8.51±0.43	7.72–9.01	p = 0.720
ONHV (mm^3^)	3.57±1.13	1.6−6.0	1.76±0.86	0.41–4.22	**p<0.001**
					B = 1.8; SE = 0.4
ONHH (µm)	0.46±0.09	0.37−0.64	0.40±0.05	0.34−0.57	p = 0.055

Abbreviations: RNFLT  =  retinal nerve fiber layer thickness; TMV  =  total macular volume; ONHV  =  optic nerve head volume; ONHH  =  optic nerve head height; SD  =  standard deviation; Min  =  minimum value; Max  =  maximum value; GEE  =  generalized estimating equation models analyses accounting for inter-eye/intra-patient dependencies; B  =  regression coefficient; SE  =  coefficient standard error; p =  p value.

In a Spearman's Rho analysis in IIH patients only, RNFLT correlated both with ONHV (r  = 0.501, p<0.001) and ONHH (r = 0.482, p = 0.003).

### Association of ICP with ONH volume and height

Lastly, we assessed the association of ONH measures and ICP. Since the pilot trial design prohibited a direct measurement of ICP next to OCT, we deployed the latest measured lumbar puncture opening pressure, weighting it for the distance of measurement as described in methods and including only patients with ICP measurements closer to 24 months from OCT (n = 11).

Using this approach, GEE with ICP as the independent variable weighted for distance of measurement and ONHV or ONHH as the dependent variable were weakly significant for ONHV (B  = 0.020, SE  = 0.007, p = 0.004) and interestingly significant for ONHH with a negative association (B  =  −0.003, SE  = 0.0005, p<0.001), meaning that IIH patients with a high ICP showed a more depressed ONHH.

## Discussion

In this pilot study we examined IIH patients and matched controls using 3D SDOCT with a novel segmentation algorithm to assess optic nerve head changes. Our main findings are: (a) ONH volume was increased in IIH vs. CG and (b) in untreated versus treated patients. (c) In contrast, neither RNFLT nor TMV differed between IIH patients and controls, although a few patients showed very high RNFLT values. (d) ONHV was weakly associated with ICP, while ONHH showed an inverse association. Finally, (e) a substantially higher proportion of IIH patients exhibited ONHV values above the 95^th^ percentile of controls (18 eyes of 11 patients, 57.9%, including all 6 untreated patients) as compared to RNFLT (7 eyes of 4 patients, 21.0%) which indicates a higher susceptibility of 3D ONH changes to alterations in ICP than RNFLT. ONH volume showed a significant increase even in those IIH patients in whom RNFLT remained normal. Thus, automated analysis of ONH shape may present a first step towards detecting even marginal changes in the composition of optic disc swelling. The necessity of quantitative evaluation of the optic disc was recently emphasized by another clinical study using transorbital sonography to follow IIH patients around lumbar puncture to create a more valuable tool for disease monitoring [32]. This study investigated especially the 2D sonographic parameters optic nerve sheath diameter (OSND), optic nerve diameter (OND) and optic disc elevation. OSND was significantly increased in IIH patients before lumbar puncture and decreased hereafter, while OND showed no differences to controls. The penetration depth of OCT disallows an evaluation of the optic disc sheath. On the other hand SDOCT could detect morphological changes of the ONH and its boundaries in more detail, because of the much higher resolution than transorbital sonography (OCT optic resolution: 7 µm axial and 14 µm lateral; sonographic resolution in millimeter range). Thus, in the future both methods with their respective advantages and limitations may complement each other in improving assessment of the optic nerve head and sheath in IIH and other conditions associated with increased ICP.

Our findings disclose several possibilities for using quantified ONHV in practice. OCT could aid in new diagnosis of IIH, providing an easy tool to quantify ONH swelling in patients with unclear symptoms. Even if high inter-individual differences in ONH shape limit usage in cross-sectional applications, assessing changes in ONH might prove highly useful in longitudinal settings: Automated 3D ONH assessment could be potentially used for monitoring treatment effectiveness or for quantifying disease progression. This aspect is supported by the higher ONHV in pharmacologically untreated patients.

Of interest in this regard is the inverse association of ONH maximal height and ICP: IIH patients with higher ICP show less pronounced ONH height than patients with lower ICP. These results suggest, that an ONH swelling process occurs more in a plane base than in height, proposing ONHV as the more meaningful parameter of ONH quantification. In addition a wall at the rim of the optic disc is a non-pathological finding also in healthy controls, which could influence the investigative power of ONHH, because the rim is also detected by the algorithm assessing ONHH. One could also speculate that this inverse association is the result from damage to the RNFL occurring during high-pressure phases. The assumption of RNFL damage as a consequence of high ICP and papilledema is supported by the fact that some of our patients showed reduced RNFL. However, due to the sometimes long interval between ICP and OCT measurements this result should be interpreted with caution.

In our study we could not approve the previously published results of RNFLT differences between IIH patients and matched controls in time domain OCT [24]. This might be explained in part by differences in study design: First, we did not involve patients with a newly confirmed IIH. The much longer disease duration and treatment of our patient cohort could have a downsizing effect on the measured RNFLT. This is supported by a follow-up study to the above-mentioned trial that identified a significant decrease in disc swelling as detected by OCT after three months of disease duration and treatment [25]. Second, peripapillary RNFLT is limited in describing optic disc swelling because it measures the boundaries of the process only indirectly. In contrast, our study directly quantified papilledema by a novel analysis algorithm, which enables the investigator to gain more detailed information about ONH swelling itself. Although our patients still had detectable changes in OCT using this ONH analysis, these changes were just not prominent enough to show in RNFLT. Third, some of the patients in this study even showed pronounced RNFL loss, leading i.e. to an AUC of <0.5. We presume, that this RNFL thinning could be a consequence of the high ICP for years, causing neuroaxonal damage and loss [33], [34].

Unfortunately it was not possible to perform ICP and OCT measurements at the same time in this pilot trial, which is also the greatest limitation of our study. The significant association of ICP with ONHV when weighting for ICP measurement time distance from OCT points towards a possible direct correlation between ICP and ONH shape. However, these results should be interpreted with care: Since scaling of the weighting variable is unknown and was thus selected arbitrarily, the used statistical model might not be applicable. Confirmation of these results is therefore highly warranted in controlled trials merging time points of ICP and OCT measurements.

Our study points out the urgent need for specialized analysis protocols for neurologic diseases using OCT. Although OCT's informative value is limited to the retina, its value in combination with powerful and specialized software for applications in neurology can be greatly improved. Overall, the detection of the optic disc volume and height makes SDOCT in combination with the newly developed segmentation algorithm a valuable tool for evaluating papilledema in IIH patients. The custom segmentation method should be further developed for and applied in follow-up trials to determine reliability, precision and ultimately diagnostic value with sensitivity and specificity. Further statistical optimization could e.g. be achieved by combining information from both eyes into differential diagnosis models.

In summary, our findings suggest ONH volume scans as a diagnostic and progression parameter in IIH with higher sensitivity to ICP changes than RNFLT. OCT is a fast and inexpensive method. A typical examination does not exceed 10 minutes per eye. Thus, OCT could promise a fast and easy alternative to repeat lumbar punctures, exercised in Europe to follow IIH patients, and could therefore ease monitoring of disease progression and treatment response. Furthermore, the methods for quantifying ONH swelling should potentially be applicable in other disorders with elevated intracranial pressure that lead to papilledema.

## References

[1] Friedman DI, Jacobson DM (2002). Diagnostic criteria for idiopathic intracranial hypertension.. Neurology.

[2] Kesler A, Hadayer A, Goldhammer Y, Almog Y, Korczyn AD (2004). Idiopathic intracranial hypertension.. Neurology.

[3] Wall M (2010). Idiopathic Intracranial Hypertension.. Neurol Clin.

[4] Kupersmith MJ, Gamell L, Turbin R, Peck V, Spiegel P (1998). Effects of weight loss on the course of idiopathic intracranial hypertension in women.. Neurology.

[5] Ko MW, Chang SC, Ridha MA, Ney JJ, Ali TF (2011). Weight gain and recurrence in idiopathic intracranial hypertension.. Neurology.

[6] Ball AK, Clarke CE (2006). Idiopathic intracranial hypertension.. The Lancet Neurology.

[7] Friedman DI, Rausch EA (2002). Headache diagnoses in patients with treated idiopathic intracranial hypertension.. Neurology.

[8] Wong GK, Poon WS Pseudotumor Cerebri: Time to Reflect on Treatment.. World Neurosurgery.

[9] Shah VA, Kardon RH, Lee AG, Corbett JJ, Wall M (2008). Long-term follow-up of idiopathic intracranial hypertension.. Neurology.

[10] Friedman DI (2004). Pseudotumor cerebri.. Neurol Clin.

[11] Wolf A, Hutcheson KA (2008). Advances in evaluation and management of pediatric idiopathic intracranial hypertension.. Current Opinion in Ophthalmology.

[12] Huang D, Swanson EA, Lin CP, Schuman JS, Stinson WG (1991). Optical coherence tomography.. Science.

[13] Costello F, Coupland S, Hodge W, Lorello GR, Koroluk J (2006). Quantifying axonal loss after optic neuritis with optical coherence tomography.. Annals of Neurology.

[14] Petzold A, de Boer JF, Schippling S, Vermersch P, Kardon R (2010). Optical coherence tomography in multiple sclerosis: a systematic review and meta-analysis.. Lancet Neurol.

[15] Burkholder BM, Osborne B, Loguidice MJ, Bisker E, Frohman TC (2009). Macular Volume Determined by Optical Coherence Tomography as a Measure of Neuronal Loss in Multiple Sclerosis.. Arch Neurol.

[16] Bock M, Brandt AU, DÃ¶rr J, Kraft H, Weinges-Evers N (2010). Patterns of retinal nerve fiber layer loss in multiple sclerosis patients with or without optic neuritis and glaucoma patients.. Clinical Neurology and Neurosurgery.

[17] Ratchford JN, Quigg ME, Conger A, Frohman T, Frohman E (2009). Optical coherence tomography helps differentiate neuromyelitis optica and MS optic neuropathies.. Neurology.

[18] de Seze J, Blanc F, Jeanjean L, Zephir H, Labauge P (2008). Optical Coherence Tomography in Neuromyelitis Optica.. Arch Neurol.

[19] Stricker S, Oberwahrenbrock T, Zimmermann H, Schroeter J, Endres M (2011). Temporal retinal nerve fiber loss in patients with spinocerebellar ataxia type 1.. PLoS ONE.

[20] Moschos MM, Tagaris G, Markopoulos I, Margetis I, Tsapakis S (2011). Morphologic changes and functional retinal impairment in patients with Parkinson disease without visual loss.. Eur J Ophthalmol.

[21] Gordon-Lipkin E, Chodkowski B, Reich DS, Smith SA, Pulicken M (2007). Retinal nerve fiber layer is associated with brain atrophy in multiple sclerosis.. Neurology.

[22] Pfueller CF, Brandt AU, Schubert F, Bock M, Walaszek B (2011). Metabolic Changes in the Visual Cortex Are Linked to Retinal Nerve Fiber Layer Thinning in Multiple Sclerosis.. PLoS ONE.

[23] Dörr J, Wernecke KD, Bock M, Gaede G, Wuerfel JT (2011). Association of Retinal and Macular Damage with Brain Atrophy in Multiple Sclerosis.. PLoS ONE.

[24] Skau M, Milea D, Sander B, Wegener M, Jensen R (2010). OCT for optic disc evaluation in idiopathic intracranial hypertension.. Graefes Arch Clin Exp Ophthalmol.

[25] Skau M, Sander B, Milea D, Jensen R (2010). Disease activity in idiopathic intracranial hypertension: a 3-month follow-up study.. J Neurol.

[26] Yri HM, Wegener M, Sander B, Jensen R (2006). http://www.springerlink.com/content/mmkj64211w254h54/.

[27] Waisbourd M, Leibovitch I, Goldenberg D, Kesler A (2007). http://www.sciencedirect.com/science/article/pii/S0303846711001661.

[28] Heidary G, Rizzo JF (2010). Use of Optical Coherence Tomography to Evaluate Papilledema and Pseudopapilledema.. Semin Ophthalmol.

[29] Strouthidis NG, Fortune B, Yang H, Sigal IA, Burgoyne CF (2011). Longitudinal Change Detected by Spectral Domain Optical Coherence Tomography in the Optic Nerve Head and Peripapillary Retina in Experimental Glaucoma.. Investigative Ophthalmology & Visual Science.

[30] Smith JL (1985). Whence pseudotumor cerebri?. J Clin Neuroophthalmol.

[31] Kadas EM, Kaufhold F, Schulz C, Paul F, Polthier K et al (2012). 3D optic nerve head segmentation in idiopathic intracranial hypertension..

[32] Bäuerle J, Nedelmann M (2011). Sonographic assessment of the optic nerve sheath in idiopathic intracranial hypertension.. Journal of Neurology.

[33] Wall M, George D (1991). IDIOPATHIC INTRACRANIAL HYPERTENSION.. Brain.

[34] Gu XZ, Tsai JC, Wurdeman A, Wall M, Foote T (1995). Pattern of axonal loss in longstanding papilledema due to idiopathic intracranial hypertension.. Curr Eye Res.

